# Chromosome-level genome assembly of the Japanese sawyer beetle *Monochamus alternatus*

**DOI:** 10.1038/s41597-024-03048-y

**Published:** 2024-02-13

**Authors:** Yong-Fu Gao, Fang-Yuan Yang, Wei Song, Li-Jun Cao, Jin-Cui Chen, Xiu-Jing Shen, Liang-Jian Qu, Shi-Xiang Zong, Shu-Jun Wei

**Affiliations:** 1 Beijing Key Laboratory for Forest Pest Control, Beijing Forestry University, Beijing, 100083 China; 2 Institute of Plant Protection, Beijing Academy of Agriculture and Forestry Sciences, Beijing, 1000097 China; 3 Key Laboratory of Forest Protection of National Forestry and Grassland Administration, Ecology and Nature Conservation Institute, Chinese Academy of Forestry, Beijing, 100091 China

**Keywords:** Entomology, Genome

## Abstract

The Japanese sawyer beetle *Monochamus alternatus* (Coleoptera: Cerambycidae) is a pest in pine forests and acts as a vector for the pine wood nematode *Bursaphelenchus xylophilus*, which causes the pine wilt disease. We assembled a high-quality genome of *M. alternatus* at the chromosomal level using Illumina, Nanopore, and Hi-C sequencing technologies. The assembled genome is 767.12 Mb, with a scaffold N50 of 82.0 Mb. All contigs were assembled into ten pseudo-chromosomes. The genome contains 63.95% repeat sequences. We identify 16, 284 protein-coding genes in the genome, of which 11,244 were functionally annotated. The high-quality genome of *M. alternatus* provides an invaluable resource for the biological, ecological, and genetic study of this beetle and opens new avenues for understanding the transmission of pine wood nematode by insect vectors.

## Background & Summary

The pine wilt disease is currently considered one of the most serious threats to pine forests worldwide^1–3^. This disease is caused by the pinewood nematode *Bursaphelenchus xylophilus* (Steiner and Buhrer) (Nematoda: Aphelenchoididae), an invasive species originally from North America^2^. The natural spread of pinewood nematode usually requires insect vectors^4^. The longhorn beetles from the *Monochamus* (Coleoptera: Cerambycidae) are the primary vectors of the pinewood nematode^5–7^. The Japanese sawyer beetle *Monochamus alternatus* (Hope) (Coleoptera: Cerambycidae: Lamiinae) is an effective vector of the pinewood nematode^8^. The *M. alternatus* can cause damage directly to various species of pine trees from the genera *Pinus*, *Cedrus*, *Abies*, *Picea*, and *Larix*^4^. This beetle is widely distributed in Japan, Korea, Laos, Vietnam, and the surrounding countries^9,10^. Single *M. alternatus* can harbor, on average, 15,000 and up to 280,000 pinewood nematodes in its tracheal system^11,12^. *Monochamus saltuarius* is another species that was reported as the vector beetle of pinewood nematode in Japan, Europe, and China. It was first reported to transmit the pinewood nematode to native *Pinus* species in Liaoning Province, China^13^. It is crucial to understand the ecology and genetics of *M. alternatus* and how it transmits pinewood nematodes^14,15^. The genome of *M. saltuarius* has been sequenced and assembled^16^. However, the genome of *M. alternatus* has yet to be determined. Bridging this knowledge gap will greatly aid our control efforts against *M. alternatus* and pine wilt disease^17^.

In this study, we assembled the chromosome-level genome of *M. alternatus* using a combination of Nanopore, Illumina short-read sequencing, and chromosome conformation capture (Hi-C) technologies to provide genomic resources for future investigations on the ecology, genetics, and evolution of the *M. alternatus* and the interaction between the pinewood nematode and its insect vector.

## Methods

### Sample preparation

Samples of *M. alternatus* were from a laboratory strain reared at the Key Laboratory of Forest Protection of National Forestry and Grassland Administration, Ecology and Nature Conservation Institute, Chinese Academy of Forestry, Beijing, China. This strain was reared for about 30 generations in the laboratory. A single female adult was used to construct libraries of Illumina short read, Oxford Nanopore Technology (ONT) long read sequencing, and Hi-C. The samples were starved for 24 hours, and the guts of the adults were removed to minimize contamination from gut microbes. In addition, we collected three larvae, pupae, and adults of *M. alternatus* for transcriptome sequencing. All samples were frozen in liquid nitrogen and stored at −80 °C until further usage.

### Genomic DNA and RNA sequencing

For short-read sequencing, genomic DNA was extracted using the QIAGEN® Genomic DNA extraction kit (Qiagen, Hilden, Germany) according to the standard operating procedure provided by the manufacturer. The pair-end library with an insert size of about 300 bp was prepared using VAHTSTM Universal DNA Library Prep Kit for Illumina® V3 (Vazyme, ND607, Nanning, China) and sequenced on the Illumina NovaSeq 6000 platform (Illumina, San Diego, CA, USA). We obtained 42.5 Gb Illumina short reads (Table 1).

**Table 1:** Library sequencing data and methods used in this study to assemble the *Monochamus alternatus* genome.

Sequencing strategy	Platform	Usage	Insertion size	Clean data (Gb)	Coverage (X)
Short-read	Illumina	Genome survey	300 bp	42.50	55
Long-read	Nanopore	Genome assembly	10–20 kb	142.7	186
Hi-C	Illumina	Hi-C assembly	300 bp	81.7	106
RNA-seq	Illumina	Anno-evidence	300 bp	18.9	27

For long-read sequencing, high molecular weight genomic DNA was isolated using the QIAGEN® Genomic DNA extraction kit (Qiagen, Hilden, Germany) according to the standard operating procedure provided by the manufacturer. A total of 3–4 μg DNA was used as input material for the ONT library preparation. Long DNA fragments were selected using the PippinHT system (Sage Science, USA). The A-ligation reaction was conducted with the NEBNext Ultra II End Repair/dA-tailing Kit (Ipswich, MA, USA). The adapter in the SQK-LSK109 (Oxford Nanopore Technologies, UK) was used for further ligation reaction. About 700 ng DNA library was constructed and performed on a Nanopore PromethION sequencer instrument (Oxford Nanopore Technologies, UK) at the GrandOmics Biosciences Co., Ltd. (Wuhan, China), and 142.7 Gb long reads were generated (Table 1).

For Hi-C sequencing, the library was prepared according to the standard protocol described by Belton with minor modifications^18^. An adult of *M. alternatus* was cut into pieces and mixed with 2% formaldehyde solution for cross-linking. Glycine (2.5 M) was added to stop this reaction, and the sample was homogenized to separate the nuclei. The purified nuclei were dissolved in SDS and incubated at 65 °C for 10 min. After quenching the SDS with Triton X-100, the sample was digested with Dpn II and marked by incubating with biotin-14-dCTP. Biotin from nonligated DNA ends was removed by T4 DNA polymerase. Then, the Hi-C library was prepared by Truseq Nano DNA HT Kit (Illumina, USA) and sequenced on the Illumina HiSeq platform with paired-end 150-bp reads (Illumina, San Diego, CA, USA) at Annoroad Gene Technology Co., Ltd. (Beijing, China). A total of 81.7 Gb (106 × coverage) of clean data was generated (Table 1).

For transcriptome sequencing, total RNA was extracted from a single *M. alternatus* (larva, pupa, and adult, respectively) using the RNAprep Pure Tissue Kit (Tiangen, China). Library was constructed using a TruSeq RNA sample preparation kit (Illumina, San Diego, CA, USA) and sequenced on the Illumina NovaSeq 6000 platform (Illumina, San Diego, CA, USA) with the paired-end mode at GrandOmics Biosciences Co., Ltd. (Wuhan, China). A total of 18.9 Gb transcriptome data was obtained (Table 1).

### Estimation of genomic characteristics

**Fig. 1:**
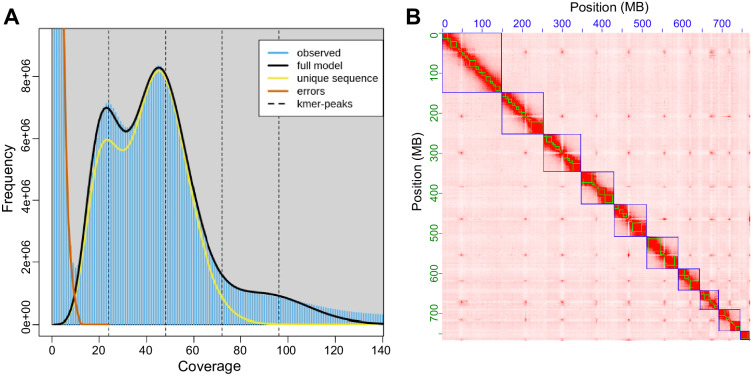
Feature estimation and assembly of *Monochamus alternatus* genome. (**A**) Estimation of *M. alternatus* genomic features. The 17-mer distributions showed double peaks: the first peak with a coverage of 100 indicates genome duplication, and the highest peak with a coverage of 200 represents a genome-size peak. *M. alternatus* genome size was calculated to be 667 Mb with heterozygosity rate of 1.31% and duplication rate of 1.55%. (**B**) **Genome-wide contact matrix of** ***Monochamus alternatus*** **generated using Hi-C data**. Each black square represents a pseudo-chromosome. The color bar indicates the interaction intensity of Hi-C contacts.

The Illumina raw reads were checked and filtered using Trimmomatic version 0.39-2^19^ to discard reads with adaptors, unknown nucleotides (Ns), or >20% low-quality bases. Genome size, heterozygosity, and duplication were estimated by using Jellyfish version 2.2.10^20^ and GenomeScope version 2.0^21^ based on the 17-mer depth distribution. The estimated genome size was 667 Mb, with a heterozygosity rate of 1.31% and a duplication rate of 1.55% (Fig. 1A).

### Genome assembly

A draft genome at contig level was assembled using NextDenovo version 1.2.5 (https://github.com/Nextomics/NextDenovo) with default parameters (genome-size = 667, read-cutoff = 3k) based on Nanopore long reads. Purge_dups was used to remove alternative haplotype and redundant fragments in the contig assembly. We performed Hi-C analysis to further anchor the assembly into chromosome-scale linkage groups. The Hi-C clean reads were cleaned using Fastp^22^ and mapped to the contigs using BWA. YaHS version 1.2a.1^23^ and Juicertools version 1.19.02^24^ were used to assemble and manual correction. As a result, 98.21% of the contigs were anchored to 10 pseudo-chromosomes, which were presented in the heatmap of the chromatin contact matrix (Fig. 1B). At last, two rounds of polishing with ONT reads and Illumina reads were performed using NextPolish version 1.4.0^25^. The output chromosome-level genome has a size of 767.12 Mb, N50 of 82.0 Mb, maximum length of 149.24 Mb, and GC content of 32.35% (Table 2).

**Table 2:** Statistics for the chromosomal-level genome of the *Monochamus alternatus*.

**Survey statistics**	Sequencing platform	Illumina
	Estimated genome size (bp)	667,998,064
	Heterozygosity rate (%)	1.31
	Duplication rate (%)	1.55
**Assembly statistics**	Sequencing platform	Illumina, Nanopore, Hi-C
	Total length (bp)	767,125,294
	Longest scaffold length (bp)	149,245,057
	Scaffold N50 (bp)	82,003,078
	GC content (%)	32.35
**Annotation statistics**	Anchored to chromosome (%)	98.21%
	Bases masked	63.95%
	Number of annotated protein-coding gene	16,284
	Number of functionally annotated gene	11,244

### Genome annotation

The protein-coding genes in the *M. alternatus* genome were predicted under three lines of evidence, including RNA-based, *ab initio*, and homology-based methods. For the RNA-based method, short transcriptome reads were mapped to the genome using Hisat2^26^. Then, the aligned BAM files were used to assemble the transcripts using Stringtie version 2.1.4^27^. The genes were predicted using PASA version 2.0.2 with default settings^28^. The *ab initio* prediction was performed using Augustus version 3.4.0^29^ and SNAP version 2006-07-28^30^. The gene models in Augustus and SNAP were trained based on transcripts longer than 300 bp generated by PASA. In the homology-based prediction, we gathered evidence of homologous genes from Coleoptera species, including *Anoplophora glabripennis*^31^, *Tribolium castaneum*^32^, *Dendroctonus ponderosae*^33^ and *Diabrotica virgifera*^34^. Redundant genes in the pooled gene set were removed using CD-HIT^35^. Maker version 3.01.04^36^ pipeline was used to perform the homology-based prediction. At last, the evidence from these methods was combined using EvidenceModeler (EVM) version 1.1.1^37^ to obtain a non-redundant consensus official gene set (OGS).

The predicted genes were functionally annotated using Eggnog-Mapper version 2.1.9^38^. Five methods were used to search against several public databases, including Gene Ontology (GO), Clusters of Orthologous Groups of Proteins (COG), Kyoto Encyclopedia of Genes and Genomes (KEGG), CAZY, and Pfam. In summary, we identified 16,284 protein-coding genes (Table 2), of which 11,244 were functionally annotated (Table 3).

**Table 3:** Number of functionally annotated protein-coding gene in different databases.

Database name	Annotated number	Percent (%)
GO	8311	73.91
KEGG	4751	42.25
COG	10635	94.58
CAZY	295	2.62
PFAM	10519	93.55
Total	11244	98.44

### Repeats prediction

Homology-based and *de novo* prediction methods were used to detect transposable elements (TEs). Briefly, repeats sequences were detected using RepeatMasker version 4.1.2 (-no_is -norna -xsmall -q)^39^, against the Repbase, Dfam database, and species-specific repeat library identified by RepeatModeler version 2.0.3. Finally, 63.95% of the genome was identified to be repeat DNA. Overall, 576,182 transposable elements (TEs), including 178,967 retroelements (189 short interspersed nuclear elements (SINEs), 144,289 long interspersed nuclear elements (LINEs), and 34,489 long terminal repeats (LTR)) and 397,215 DNA transposons were identified. Five hundred twenty-three satellites and 678 simple repeats were identified as tandem repeats (TRs), accounting for 0.03% of the *M. alternatus* genome (Table 4).

**Table 4:** Repeats elements statistics in genome of *Monochamus alternatus* using RepeatMasker.

Item	Number of element	Length occupied (bp)	Percentage of sequence (%)
Retroelements	178,967	76,112,632	9.72
SINEs	189	14,122	0
LINEs	144,289	56,571,154	7.23
LTR elements	34,489	19,527,356	2.49
DNA transposons	397,215	133,929,988	17.11
Rolling-circles	7,406	2,474,246	0.32
Unclassified	1,235,289	287,908,114	36.78
Total interspersed repeats	NA	497,950,734	63.61
Small RNA	0	0	0
Satellites	523	126,782	0.02
Simple repeats	678	105,396	0.01
Low complexity	0	0	0

SINEs: short interspersed nuclear elements; LINEs: long interspersed nuclear elements; LTR: long terminal repeat.

### Non-coding RNA annotation

For non-coding RNA annotation, the transfer RNA (tRNA) was annotated by tRNAscan-SE version 1.3.1 based on the structural characteristics of tRNA^40^, whereas the ribosome RNA (rRNA) was predicted by RNAmmer version 1.2^41,42^. We obtained 498 tRNA and 107 rRNA genes, including 98 8s_rRNA, five 28s_rRNA, and four 18s_rRNA genes in the *M. alternatus* genome (Table 5).

**Table 5:** Statistics of non-coding RNAs in genomes of *Monochamus alternatus*.

Class	Type	Count
rRNA count	8s_rRNA	98
	28s_rRNA	5
	18s_rRNA	4
Cove stats	Candidate tRNAs read	9436
	Cove-confirmed tRNAs	498
	Bases scanned by Cove	987,197
	Seq scanned by Cove	0.1%
tRNA count	tRNAs decoding Standard 20 AA	452
	Selenocysteine tRNAs (TCA)	0
	Possible suppressor tRNAs (CTA, TTA)	2
	tRNAs with undetermined/unknown isotypes	3
	Predicted pseudogenes	41
Total tRNAs		498
tRNAs with intron		43

## Data Records

The genome project was deposited at NCBI under BioProject number PRJNA819115. Illumina sequencing data for genome survey were deposited in the Sequence Read Archive at NCBI under accession number SRR26115523^43^. Hi-C sequencing data were deposited in the Sequence Read Archive at NCBI under accession number SRR26146338^44^. Nanopore sequencing raw data were deposited in the Sequence Read Archive at NCBI under accession number SRR26157698^45^. RNA-seq data were deposited in the Sequence Read Archive at NCBI under accession numbers SRR26116071- SRR26116073^46–48^. The final chromosome assembly, genome structure annotation, amino acid sequences and functional annotation results of protein-coding genes were deposited to Figshare repository under a DOI number of 10.6084/m9.figshare.c.6849162.v1^49^. The final chromosome assembly was deposited in GenBank under accession number JAYMDT000000000^50^.

## Technical Validation

The Hi-C heatmap exhibits the accuracy of genome assembly, with relatively independent Hi-C signals observed between the ten pseudo-chromosomes (Fig. 1B). We assessed the accuracy of the final genome assembly by mapping Illumina short reads to the *M. alternatus* genome with BWA-MEM2 version 0.7.1721^51^. The mapping rate for Illumina reads was 98.71%. The findings indicate that the quality of our assembled genome is high.

To assess the completeness of genome assembly and OGS, we run Benchmarking Universal Single-Copy Orthologues (BUSCO version 5.2.2) using the insecta_odb10 database, which contains 1367 conserved genes^52^. For Contig-level, in the first round, the BUSCO analysis showed that 93.8% (single-copied gene: 93.2%, duplicated gene: 0.6%) of 1367 single-copy genes were identified as complete, 3.3% of genes were fragmented, and 2.9% of genes were missing in the assembled genome. For the chromosome-level assembly, BUSCO analysis showed that 99.7% (single-copied gene: 99.0%, duplicated gene: 0.7%) of 1367 genes were identified as complete, 0% of genes were fragmented, while 0.3% of genes were missing in the assembled genome. For OGS, BUSCO analysis showed 96.7% completeness, with only 0.5% of genes duplicated, 1.5% fragmented, and 1.8% missing (Table 6).

**Table 6:** BUSCO evaluation of genome assemblies.

Description	Contig-level		Chromosome -level		Official gene set (OGS)	
	Number	Percent (%)	Number	Percent (%)	Number	Percent (%)
Complete gene (C)	1282	93.8	1363	99.7	1322	96.7
Complete and single-copy gene (S)	1274	93.2	1353	99	1315	96.2
Complete and duplicated gene (D)	8	0.6	10	0.7	7	0.5
Fragmented gene (F)	45	3.3	0	0	21	1.5
Missing gene (M)	40	2.9	4	0.3	24	1.8
Total gene searched	1367	/	1367	/	1367	/

## Data Availability

There were no custom scripts or code utilized in this study.
